# Thermoelectric properties of SnSe nanowires with different diameters

**DOI:** 10.1038/s41598-018-30450-5

**Published:** 2018-08-10

**Authors:** Jose A. Hernandez, Angel Ruiz, Luis F. Fonseca, Michael T. Pettes, Miguel Jose-Yacaman, Alfredo Benitez

**Affiliations:** 1 Department of Physics, University of Puerto Rico, Rio Piedras Campus, San Juan, PR 00931 USA; 2 Department of Mechanical Engineering and Institute of Materials Science, University of Connecticut, Storrs, CT 06269-3139 USA; 3 Department of Physics, University of Texas, San Antonio Campus, San Antonio, TX 78249 USA; 4 Present Address: Center for Integrated Nanotechnologies (CINT), Materials Physics and Applications Division, Los Alamos National Laboratory, Los Alamos, New Mexico 87545 USA

## Abstract

Tin selenide (SnSe) has been the subject of great attention in the last years due to its highly efficient thermoelectricity and its possibilities as a green material, free of Pb and Te. Here, we report for the first time a thermoelectricity and transport study of individual SnSe micro- and nano-wires with diameters in the range between 130 nm and 1.15 *μ*m. X-ray diffraction and transmission electron microscopy analyses confirm an orthorhombic SnSe structure with Pnma (62) symmetry group and 1:1 Sn:Se atomic ratio. Electrical and thermal conductivity and the Seebeck coefficient were measured in each individual nanowire using a specialized suspended microdevice in the 150–370 K temperature range, yielding a thermal conductivity of 0.55 *Wm*^−1^ *K*^−1^ at room temperature and *ZT* ~ 0.156 at 370 K for the 130 nm diameter nanowire. The measured properties were correlated with electronic information obtained by model simulations and with phonon scattering analysis. The results confirm these structures as promising building blocks to develop efficient temperature sensors, refrigerators and thermoelectric energy converters. The thermoelectric response of the nanowires is compared with recent reports on crystalline, polycrystalline and layered bulk structures.

## Introduction

Due to the increasing use and costs of fossil fuels and to the contamination that conventional energy production is causing, a worldwide effort is underway to find alternative sources of energy that can replace traditional ones. A viable energy conversion technology should offer energy efficiency comparable to actual technology and with competitive production costs^1^. With the discovery of new materials, thermoelectricity is getting increasing interest in the last years as a green alternative for energy production that can use available heat waste from a wide variety of systems, such as automobile engines and solar heating panels^2–5^. The thermoelectric conversion efficiency is characterized by the figure of merit (ZT). For a particular material, ZT can be expressed in terms of its Seebeck coefficient (S), total thermal conductivity (*κ*) and electrical conductivity (*σ*) as *ZT* ≡ *S*^2^*σT*/*κ*. For years research has focused on many different aspects of electronic band structure engineering and thermal conductivity minimization strategies, including finding new materials with larger S values^5–7^. Insulators show high S but their electrical conductivities are too small. Metals, on the other hand exhibit large *σ* but their Seebeck coefficients are too small. Semiconductors are materials of choice for this application because they combine relative high S values with moderate electric conductivities.

The increasing of *ZT* for bulk materials has been limited by the fact that *S*, *κ*, and *σ*, are interdependent. On one hand, an increasing density of free charge carriers that can increase *σ*, reduces *S* and increases *κ*. On the other hand, the increase of *σ* without decreasing *S* requires reducing the density of crystal defects, which will increase *κ* as well. It was suggested that this interdependence can be bypassed using low-dimensional structures such as quantum dots and nanowires, where phonon-boundary scattering reduces *κ* without changing the properties of the material at the scale of the electron’s scattering events; at the same time the asymmetric density of states near the Fermi level in nanostructures can increase *S*^8–11^. Following this idea, a *ZT* = 2 at room temperature was reported in 2002 for a quantum-dot superlattices^12^. In 2012, a *ZT* value of ~2.2 at 915 K was reported in a heterostructure, where phonon scattering at various length scales was promoted by introducing a hierarchical architecture design in a nanostructured PbTe-based system^13,14^. The explanation for the high ZT is the structure of the system’s boundaries at the atomic, nano and meso, scales that is capable of producing efficient scattering events for phonons over abroad range of wavelengths. Recent calculations in 1D-like nanostructures support significant reduction of the thermal conductivities due to size confinement, enhanced anisotropy effects and interfaces in materials such as diamond^15,16^, GaSb^17^, InAs^18,19^, InSb, InP and GaAs^19^. In 2014 Zhao *et al*.^20^, reported a record figure of merit, *ZT* ~ 2.6 at 923 K along the *b*–axis of single crystalline SnSe. This chalcogenide material adds an extra advantage in its composition – it is lead free and shows ultralow lattice thermal conductivity due to its layered structure, with atoms arranged similarly to a highly distorted rock salt structure, where the bonding of SnSe interacts covalently within the layers and forms anharmonic and anisotropic bonding^7,21,22^; along the efforts to optimize this efficiency, highly doped *p*–type SnSe materials have been studied but their increasing charge density leads to a decrease in the Seebeck coefficient and in the figure of merit, with a maximum value of *ZT* = 0.6 reported at 1023 K with *Ag*–doping^23^, 2.0 in hole-doped crystals at 773 K^24^, and below 1 for Pb, Cu, Al, In and Ag doping^25^ at *T* > 750 K. Many theoretical calculations have been carried out to describe the relationship between structural, electronic, phononic and thermoelectric properties in orthorhombic materials, and specially in *n*– and *p*–type SnSe^26–30^ and in single monolayers^31^. Calculations suggest the possibility of a high thermoelectric figure of merit in nanostructured SnSe crystals^32^, yet neither individual nanostructures or the diameter dependence of thermoelectric performance in SnSe nanowires has been reported.

## Results and Discussion

Here we report the temperature and diameter dependence of the electrical, thermal, and thermoelectric properties of SnSe nanowires. The synthesis of the SnSe nanowires was carried out using a catalyst-assisted thermal vapor-liquid-solid (VLS) process, similar to the procedure used by Butt *et al*.^33^, in which high purity powders of tin (99.998% purity, Sigma-Aldrich) and selenium (99.995%, Sigma-Aldrich) were used as source materials. The mixed powders were loaded on alumina boats and positioned at the center of the first hot zone of a two-zones horizontal quartz tube furnace (MTI 1200X). Several sapphire substrates were coated with a thin Au film with thickness between 1–3 nm, were located in the middle of an alumina boat positioned between both heating zones. The furnace was heated to 860 °C and 550 °C in the precursor and substrate zone respectively, at a rate of 10 °C min^−1^ and the temperature was then maintained constant for 60 min, before natural cooling to room temperature. During the synthesis the system was kept at atmospheric pressure, using 100 sccm of Ar as the carrier gas.

**Figure 1:**
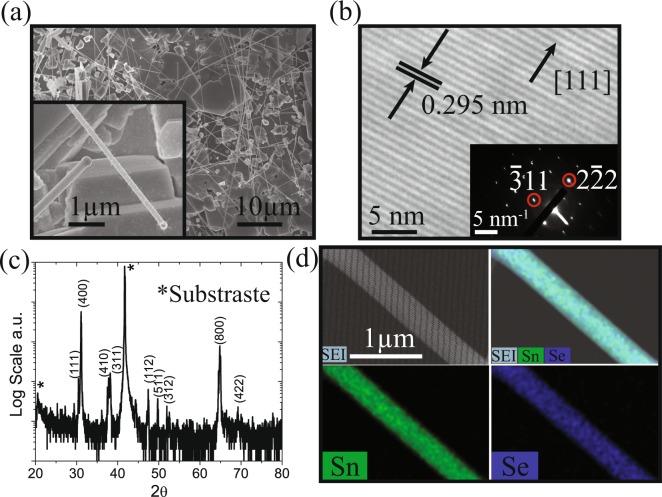
(**a**) Scanning electron microscopy (SEM) image of the as-grown SnSe nanowires. (**b**) Phase contrast transmission electron microscopy image and (b, inset) selected area electron diffraction pattern for a measured SnSe NW (1.15 *μ*m diameter). (**c**) X-ray diffraction pattern (*corresponds to the sapphire substrate). (**d**) SEM energy dispersive X-ray spectroscopy mapping for a measured SnSe nanowire (480 nm diameter).

The SnSe nanowires were formed uniformly on the surface of the substrates, with different diameters and several tens of microns in length. Figure 1a shows the scanning electron microscopy (SEM) image of as deposited nanowires. The higher magnification inset shows a typical nanowire grown from a SnSe seed crystal using Au as the catalyst. Transmission electron microscopy (TEM) analysis (Fig. 1b) shows high crystalline order at the scale of the image of the measured nanowires and gives an interplanar spacing value of *d*_[111]_ = 2.9501 *Å* corresponding to the array of planes perpendicular to the nanowire axis consistent with a SnSe crystal growth along the $[111]$ direction. This information is supported by selected area electron diffraction and X-ray diffraction (XRD) patterns that confirm the formation of orthorhombic phase (Pnma) with lattice parameters: *a* = 11.46 *Å*, *b* = 4.14 *Å* and *c* = 4.38 *Å*, *α* = *β* = *γ* = 90°, where *d*_[111]_ = 2.942 *Å*. XRD (Fig. 1c) shows only SnSe patterns without any evidence of crystalline SnO, SnO_2_, SnSe_2_ or Sn and Se byproducts. Quantified data from SEM energy dispersive X-ray spectroscopy (EDS) is consistent with an approximate 1:1 Sn:Se atomic ratio and compositional uniformity, a slightly reduced Sn signal could be evidence of native Sn vacancies in our *p*–type samples (the EDS spectra and quantification is given in Figure S1 of the Supplementary Information).

**Figure 2:**
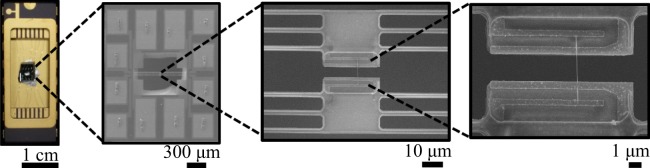
From left to right, optical image of the micro-thermometry device wired to a chip carrier, followed by low and high magnification SEM images of a 130 nm diameter SnSe nanowire transferred onto the microdevice.

After synthesis, the nanowires were detached from the substrates by sonication in ethanol and dispersed on a Si substrate for inspection and micromanipulation. Nanowires with different diameters were selected and transported to suspended microdevices to measure *S*, *σ*, and *κ*, according to the procedure detailed in previous studies^34–37^, (see Supplementary Information, Figure S2 for experimental measurement method details). Figure 2 shows a close-up SEM image of a typical SnSe nanowire integrated onto the measuring microdevices. A focused ion beam system (JEOL JEM9310 FIB) was used to remove possible contaminants from the surface of the nanowire at the nanowire/electrode regions, and to deposit Pt at these sites in order to establish a good electrical contact. The possible defect formation due to Ga^+^ ion bombardment during FIB deposition was kept to a minimum by avoiding unnecessary Ga^+^ beam irradiation of the entire nanowire but slight damage cannot be excluded completely. After the transport measurements, each nanowire was observed via SEM and TEM to determine diameters and lengths among the electrodes such that conductivities could be obtained from the corresponding measured conductances. (See Supplementary Information, Figures S3 and S4 for SEM images of all 7 measured nanowires and their measured thermal conductances, respectively).

**Figure 3:**
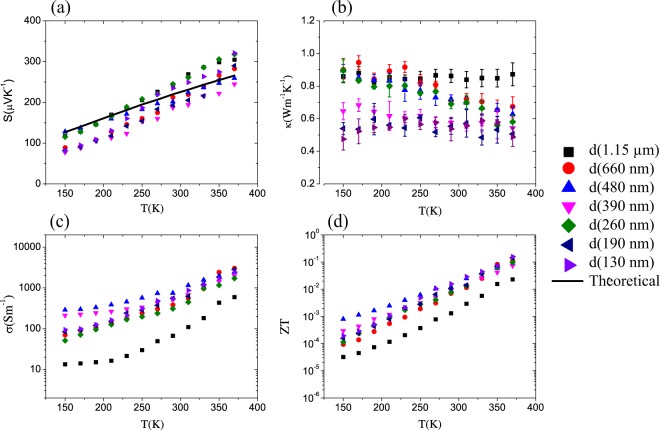
(**a**) Measured Seebeck coefficient S and theoretical calculations for a free charge concentration of 10^19^ cm^−3^, (**b**) thermal conductivity *κ*, (**c**) electrical conductivity *σ* and (**d**) *ZT* of SnSe nanowires with different diameters as a function of temperature. Uncertainties in the measurements of S are too small to be noticed in the plot.

The measured Seebeck coefficient, thermal conductivity, electrical conductivity, and calculated figure of merit as a function of temperature of nanowires with different diameters are shown in Fig. 3. In all cases the measured *S* was positive indicating *p*–type transport, increased in magnitude with increasing temperature, and did not show a clear diameter dependence. These results are in overall agreement with the study by Zhao *et al*.^20^ of bulk SnSe and SnSe doped crystals^14,24^, Singh *et al*.^25^ and Sassi *et al*.^38^ of bulk polycrystalline samples, and with the studies of nanostructured SnSe sheets by Serrano-Sanchez *et al*.^39^ Fig. 3b confirms ultralow thermal conductivities for all nanowires and is in the same order of the reported values for bulk samples^20,24,38^. *κ* values were corrected to consider the thermal contact resistance of the measuring device. The correction considers the interfacial area in each sample and interfacial adhesion energy^32,40^ explained in detail by Mavrokefalos *et al*.^35^.

A significant reduction in *κ* is observed in the samples with smaller diameters. In the thicker nanowires, *κ* slightly decreases when the temperature is increased as found for the bulk samples in Zhao *et al*.^20^ for smaller diameters (130 nm, 190 nm and 390 nm) the thermal conductivity remains practically constant or with a small increase with temperature in the 130 nm wire. This behavior is consistent with the increasing contribution of phonons surface scattering in thin nanowires^37,41,42^ although we note that a recent thermal conductivity accumulation study by Guo *et al*.^32^ found negligible contribution to the lattice thermal conductivity of phonons with wavelengths above ~100 nm. Thus, both the temperature dependence of the thermal conductivity and the magnitude indicate static scattering processes are considerable in the samples of this work. From Guo *et al*.^32^ work, we should expect that anharmonic processes will dominate in nanowires of the diameters considered here. The *d* = 1.15 *μm* sample shows reduced temperature dependence in the measured temperature range and significantly lower electrical conductivity when compared with other samples, suggesting a higher density of crystal defects in this particular nanowire.

The electrical resistance measurements were performed in a four-point configuration and the electrical conductivity was calculated after the determination by TEM and SEM of each nanowire’s length and diameter (see Figures S3 and S5, Supplementary Information). Figure 3c shows that *σ* is thermally activated in all samples within the measured temperature range as has been reported for polycrystalline SnSe^23,25,38,39,43^ and consistent with unintentional *p*–doping of the nanowires attributed to native Sn vacancies that act as double acceptors. Figure 3c shows variations of *σ* between samples at low temperature (T ~ 150 K) with no clear dependence on diameter but at higher temperatures these differences become less significant, reaching values in the order of 1000 *Sm*^−1^ at room temperature, consistent with reports in bulk polycrystalline samples^23,25,38^. In the event that carrier lifetimes are dominated by boundary scattering a correlation between the nanowire diameter and *σ* at low temperature is expected. However, in our samples the carrier concentration remains roughly constant for all samples as the thermopower is roughly sample independent. Thus, the presence of other minor impurities or point defects can lead to the order of magnitude variation in electrical conductivity between samples at low temperature, as shown in Fig. 3c. This is also supported by the lattice thermal conductivity given in Figure S8 in the Supplementary Information, where the trend of decreasing thermal conductivity with decreasing temperature below 200 K in the two smallest diameter samples, and the nearly constant thermal conductivity of the 660 nm diameter sample, are indicative of the influence of significant phonon-impurity scattering in the samples. This can be an indication of non-negligible point defect scattering in these samples, which is supported by the low thermopower measured in the unintentionally degenerately p-doped nanowires.

Finally, we calculated the figure of merit (ZT) of each nanowire from its measured *S*, *κ* and *σ* as a function of temperature. Figure 3d shows increasing ZT for all nanowires as the temperature is increased. The maximum measured ZT was 0.156 corresponding to the thinnest nanowire at 370 K, well above what has been reported for unintentionally doped polycrystalline bulk samples^23,25,38^.

**Figure 4:**
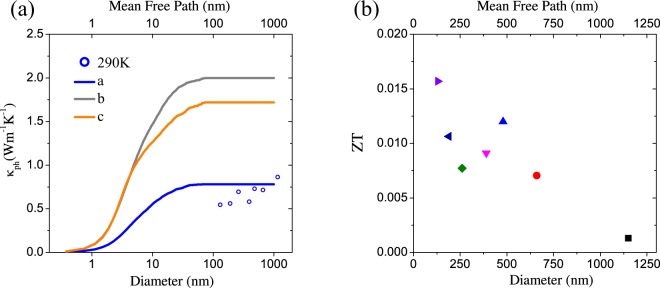
(**a**) Lattice thermal conductivity in comparison with calculated accumulated thermal conductivity data of Guo *et al*.^32^ in *a*–, *b*– and *c*–directions and (**b**) figure of merit ZT at 290 K for different diameters.

In order to gain insight into the effects of the reducing nanowire diameter on the thermal conductivity and *ZT*, Fig. 4 shows the calculated lattice thermal conductivity (*κ*_*ph*_) and the figure of merit close to room temperature (290 K) as a function of the nanowire diameter, respectively. The lattice thermal conductivity was obtained from the total thermal conductivity as *κ*_*ph*_ = *κ* − *κ*_*e*_, where *κ*_*e*_ is the electrical thermal conductivity contribution, calculated using DFT theory and the transport properties using BoltzTraP package. Figure 4a (open circles) shows an overall decrease of the thermal conductivity with decreasing diameter attributed to a reduced averaged phonon mean free path with *κ* ~ 0.55 *Wm*^−1^ *K*^−1^ for *d* = 130 nm nanowire at T = 300 K. This value is almost one half of the value reported in other bulk polycrystalline samples^23,38^ but is roughly the same order of magnitude as *κ* along the *a*–axis (continuous lines are results from calculations in reference^32^). This can be expected as the growth direction of our nanowires is oblique to this axis such that the weak bonding will reduce thermal conductivity similar to if the growth direction was along the *a*–axis. The reduction in our measured *κ* at small diameters may indicate static defects, or notably, an underestimation of the contribution of long wavelength phonons in the calculation of Guo *et al*.^32^ Fig. 4b shows the dependence of *ZT* on the diameter at room temperature in which an overall increase in *ZT* is observed by reducing the nanowire’s diameter. Figure 4b confirms a significant enhancement of *ZT* as the diameter of the nanowire is reduced below 190 nm.

## Theoretical Methods

In order to better understand the observed transport response in our SnSe nanowires, we performed density functional theory (DFT) calculations using the Vienna Ab initio Simulation Package (VASP)^44^. The many-body GW approximation was implemented with the projector augmented wave (PAW) scheme^45^, and the generalized gradient approximation of Perdew, Burke and Ernzerhof (GGA-PBE)^46^ for the electronic correlation functional. The energy cut-off for the plane wave expansion was 450 eV. With this cut-off energy we obtained a convergence on the ground state energy value of the order of 10^−6^. The Brillouin zones of the bulk SnSe were sampled in a gamma-centered grid with a k-point mesh of 5 × 15 × 15. To obtain an accurate DOS, the k-point mesh of the non-self-consistent calculation was increased to 11 × 33 × 33. Atomic positions and unit cell vectors were relaxed until all the forces and components of the stress tensor are below 0.001 eV *Å*^−1^. We used bulk parameters for the calculations because the diameters of the measured nanowires are greater than 100 nm and our calculations show a bulk-like electron energy states configuration in nanowires with diameters above 10 nm (see Figure S6 Supplementary Information).

Starting from the experimental orthorhombic structure (space group Pnma 62) with lattice parameter (*a* = 11.46, *b* = 4.14 and *c* = 4.38 *Å*) the calculated indirect bandgap (Eg) was 0.654389 eV, which is smaller than the experimental bandgap^20^. This underestimation is a well-known deficiency of the semi-local GGA approximation. When carrying out the transport calculations we corrected for this by manually expanding the gap to the experimental value. We emphasize that this correction does not alter the value of dispersive quantities (group velocity, effective masses) used in the transport calculations^47^ (Figure S7 in the Supplementary Information shows the crystal structure, the band diagram, and density of states for SnSe). These calculations served as inputs to determine the electronic transport parameters.

The transport properties were calculated based on the Boltzmann transport equation under the rigid band and constant relaxation time approximations as implemented on the BoltzTraP package^48^. This numerical package has been previously used to study SnSe materials with good results^24,26,29,31,37,49^. BoltzTraP package calculated the Seebeck coefficient S, the electrical conductivity *σ* and thermal conductivity *κ*_*e*_ according to the following expressions:

1 $$\sigma (\mu ,T)={e}^{2}\,\int \,(-\frac{\partial {f}_{0}}{\partial E})\tau (E,T)\,{\rm{\Xi }}\,(E)dE$$

2 $$S(\mu ,T)=e{\sigma }^{-1}\,\int \,(-\frac{\partial {f}_{0}}{\partial E})\,(\frac{E-\mu }{T})\,\tau (E,T)\,{\rm{\Xi }}\,(E)dE$$

3 $${\kappa }_{e}(\mu ,T)=\frac{1}{{e}^{2}T}\,\int \,(-\frac{\partial {f}_{0}}{\partial E})\,{(E-\mu )}^{2}\tau (E,T)\,{\rm{\Xi }}\,(E)dE$$

where, ${\rm{\Xi }}(E)={\sum }_{k}\,{\overrightarrow{v}}_{k}{\overrightarrow{v}}_{k}\delta (E-E(k))$; ${\overrightarrow{v}}_{k}={\hslash }^{-1}{\nabla }_{k}E(k)$ is the transport distribution function, *μ* is chemical potential, *e* is the electronic charge, *τ* is the relaxation time, *f*_0_ is the fermi function, *v*_*k*_ is the group velocity and *δ*(*E* − *E*(*k*)) is the delta function.

**Figure 5:**
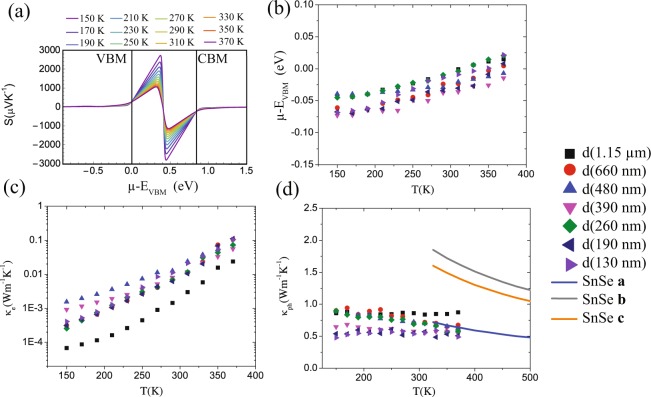
Calculated thermoelectric properties of SnSe. (**a**) Seebeck coefficient as a function of the chemical potential (*μ*-E_*VBM*_), the energy position of the band edges (E_*VBM*_ and E_*CBM*_) is identified by vertical dotted lines. (**b**) The calculated temperature dependences of the chemical potential (*μ* − *E*_*VBM*_) obtained from (a) and the measured Seebeck coefficient. (**c**) The calculated electron (*κ*_*e*_) and (**d**) lattice (*κ*_*ph*_) thermal conductivity contributions. Calculated *κ*_*ph*_ from Guo *et al*.^32^ is shown for *a*, *b* and *c* directions (solid lines).

Due to the SnSe crystal structure, the transport is anisotropic and averaged values corresponding to the $[111]$ direction are shown. Figure 5a shows the calculated S at different temperatures as a function of the chemical potential referenced to the valence band maximum, *μ* − *E*_*VBM*_. By interpolating the experimental data we determined the temperature dependence of *μ* in our samples. Figure 5b shows an increasing *μ* with temperature with values suggesting a high density of holes that keeps the chemical potential near or in the valence band at low temperatures.

To confirm these observations, BoltzTraP calculations were performed to estimate the density of holes in the material. In Fig. 3a the calculated *S*(*T*) curve for a density of holes *p* = 1 × 10^19^ cm^−3^ is included which fits the measured S for the 480 nm nanowire, thus assigning a larger density of vacancies produced during the nanowire formation as compared with other synthesis methods used to grow crystalline and polycrystalline bulk samples^20,23,38,39,43^. The electrical conductivity of the nanowires (*σ* ~ 400 *Sm*^−1^ at T = 300 K) is lower than the reported values in high quality SnSe bulk crystals with similar density of holes^24^ suggesting a larger density of defects.

As for the thermal transport, the measured thermal conductivity (*κ*) includes contributions from phonon and free charges. The calculated values of *μ* were used to determine the contribution of the charge carriers to *κ*. Equation 1 states that the conductivity is dependent of the relaxation time *τ*, therefore using the experimental conductivity one can calculate *τ*(*T*). Hence results can be interpolated and the electronic thermal conductivity *κ*_*e*_ can be obtained as function of experimental temperature using equation 3. From there, the thermal conductivity due to phonons was obtained by subtraction of the calculated electronic contribution to the measured thermal conductivity, that is to say: *κ*_*ph*_ = *κ* − *κ*_*e*_ (Fig. 5d). As shown in Fig. 5c,d, the contribution due to free charges is in some cases up to 2 orders of magnitude lower than the contribution due to phonons therefore, the measured thermal conductivity (Fig. 3b) is dominated by phonon transport (Fig. 5d) as previously observed in nanowires of other semiconductor materials^22,37^. For comparison, Fig. 5d also shows continuous lines corresponding to the calculation results of Guo *et al*.^32^. A fitting of the experimental results within the Debye Callaway approximation that considers surface scattering due to reducing diameter while keeping the density of defects constant is included in Supplementary Information (Figure S8) for comparison. In Figure S8, Supplementary Information, the two smallest diameter samples have thermal conductivity below the Debye-Callaway model calculation at temperatures below 200 K, with the trend of decreasing thermal conductivity with decreasing temperature. This can be an indication of non-negligible point defect scattering in that temperature regime, which is supported by the low thermopower measured in the unintentionally degenerately *p*-doped nanowires. Given the fact that size-dependence or sample-to-sample variation in thermopower for the different nanowires in this study is not observed is a valid approach to reduce the number of adjustable parameters in this study. Other surface effects will become observable at much smaller length scales than the focus of this report^50,51^.

## Conclusions

We have reported the VLS synthesis of SnSe nanowires and successfully measured the Seebeck coefficient and the thermal and electrical conductivities of individual nanowires with diameters from ~130 nm to ~1.15 *μm*, over a 150–370 K temperature range. HRTEM and XRD analyses confirm large crystalline grain size and growth along the $[111]$ direction. The thermoelectrical measurements were carried out with suspended specialized four-probe micro-thermometry devices. All measurements were consistent with previous publications for the material in bulk, but a clear reduction of the thermal conductivity and a clear increase in *ZT* is observed with decreasing nanowire diameter. We observed no significant dependence of the thermopower on the diameter of the nanowires within the studied range. Theoretical band structure and transport model calculations were used to correlate free charge parameters with measured data. The chemical potential values confirm high p-type doping in all cases with a hole density in the order of ~10^19^ cm^−3^. The electrical conductivity is thermally activated as reported for bulk samples and was attributed to changes in the density of holes and increasing hole mobility due to charged defects. The measured thermal conductivity in thicker nanowires is consistent with bulk data and the decrease of *κ*_*ph*_ with decreasing diameters is consistent with the corresponding increase of the phonon-surface scattering and may indicate an underestimation by previous theoretical models of the contribution of long wavelength phonons to the lattice thermal conductivity in SnSe, or an increasing density of defects in thinner nanowires. A maximum *ZT* of 0.156 at 370 K was determined for the thinnest nanowire that encourages the synthesis of nanowires with lower diameters that can be used as building blocks for high ZT nanodevices operating near room temperature.

Given the record thermoelectric ZT reported for bulk SnSe, this study in nanowires could indicate possible missing information from currently proposed theoretical models for the contributions of specific phonon mean free paths to the thermal conductivity in this material. In general, the thermoelectrical response of the materials in nanowire form brings reduced figure of merit as compared with bulk materials due to the difficulty to control with any precision their carrier concentration and crystalline quality. However, it gives insight into size effects of well characterized materials and yields unique conclusions relevant to the design of hierarchically scaled bulk materials and composites.

## Electronic supplementary material

Supplementary Information
