# Supplementary material for: Thermoelectric properties of SnSe nanowires with different diameters
Source: Sci Rep. 2018 Aug 10;8:11966. doi: 10.1038/s41598-018-30450-5 (PMC6086875; doi:10.1038/s41598-018-30450-5)
Supplement: Supplementary file 1 — Supplementary Information [file 41598_2018_30450_MOESM1_ESM.pdf]

Supplementary Information: Thermoelectric Properties of SnSe Nanowires with Different Diameters

Jose A. Hernandez[1], Angel Ruiz[1], Luis F. Fonseca[1*], Michael T. Pettes[2], Miguel Jose-Yacaman[3], and Alfredo Benitez[3]

1. Department of Physics - University of Puerto Rico - Rio Piedras Campus, San Juan PR 00931, USA.
2. Department of Mechanical Engineering and Institute of Materials Science, University of Connecticut, Storrs, CT 06269-3139, USA.
3. Department of Physics - University of Texas - San Antonio Campus, San Antonio TX, 78249, USA.

∗ luis.fonseca@upr.edu

Sample Preparation on microdevice

Once the nanowires are synthesized they are detached from the substrates by sonication in ethanol and each nanowire selected and integrated to the measuring device by micromanipulation as follows: a drop of the nanowires suspension is put on a Si wafer surface and, after alcohol evaporation, a nanowire on the Si wafer surface is selected with a tungsten nanotip attached to a Narishige micromanipulator, using a high magnification optical microscope (Nikon Eclipse E600). The selected nanowire is then attached to the tip by van der Waals forces, moved to the device, and aligned properly to let the nanowire in contact with the four Pt electrodes. Once the nanowire is in place, a focused ion beam system (Jeol JEM 9310 FIB) is used to remove possible contaminants from the surface of the nanowire and to deposit Pt at the contact sites. The measuring device with the integrated nanowire is then wire-bonded (Kulicke & Soffa 4524) to a chip carrier in order to connect it to the measurement setup.

Crystal and Chemical Characterization

The crystal structure, morphology and chemical composition of as-synthesized SnSe NWs were characterized by x-ray diffraction - XRD (Rigaku, Cu-K_α radiation with λ =1.5406 $\AA$), scanning electron microscopy - SEM (JEOL 7500F), and SEM (Hitachi 5500) equipped with an energy dispersive X-ray spectrometer (EDS) (see figure S1). High resolution transmission electron microscopy HR-TEM (JEOL 2010F) was used for high resolution imaging and SAED of one measured sample.

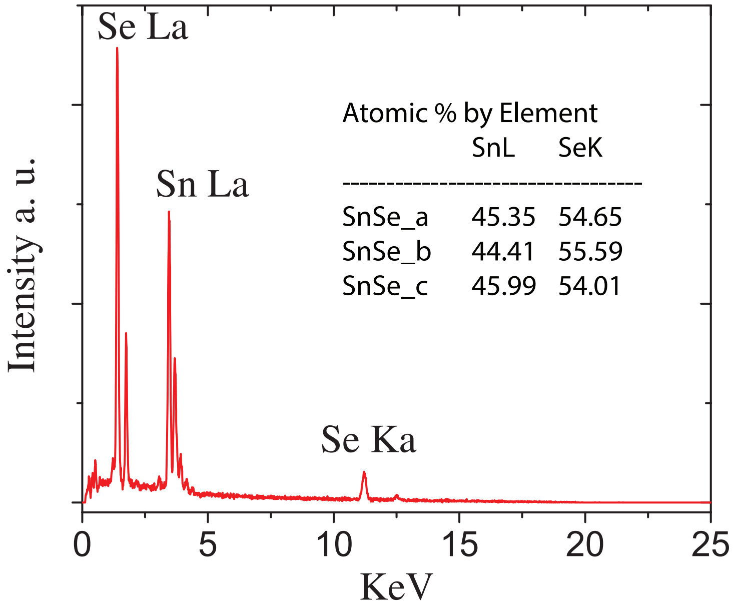

Figure S 1: SEM - EDS spectra for 3 SnSe nanowire samples. The inset shows the atomic ratio for Sn and Se

The lattice parameters were calculated by calibrating peak positions obtained from XRD and HRTEM-SAED data. XRD patterns of the as-prepared SnSe nanowires show that all diffraction peaks can be indexed to (111), (400), (311), (112), (410) and (800) planes of the orthorhombic crystal structure of SnSe (JCPDS 32-1382).

Sample Measurements

The prepared samples were placed in a cryostat chamber, pumped with a turbomolecular pump to reduce pressure to $\sim 5 \times 10^{-6}$ Torr to minimize convection heat losses. Using two locking amplifiers, a DC source, two voltage and one current amplifiers, a thermal gradient were created between the ends of the nanowire and the voltage drop was measured between the inner and outer contact electrodes, respectively. The temperatures at the ends and the two thermoelectric voltages were measured for different ambient temperatures between 150 K and 370 K using liquid Nitrogen and a temperature controller.

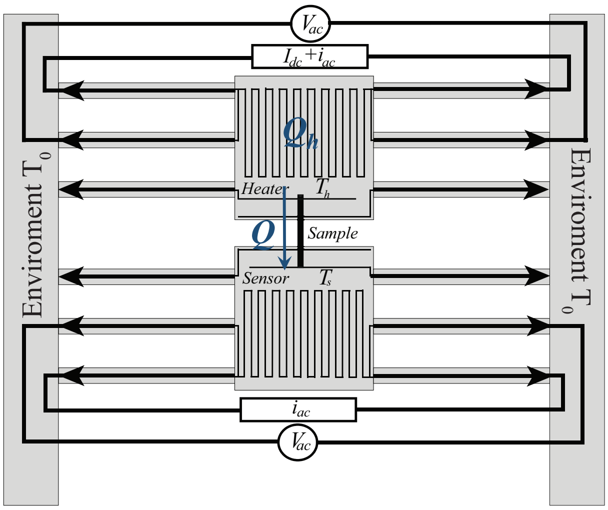

Figure S 2: Schematic of the experimental method for thermal conductance measurements of individual suspended SnSe nanowire

From these measurements the contact thermal resistance, corrected thermal conductivity, the Seebeck coefficient, and the electrical conductivity of each nanowire were obtained. The schematic of the measuring setup is shown in figure S2. Figure S3 shows SEM images for 7 measured nano- and micro-wires. Figure S4 shows the measured total thermal conductance of each sample.

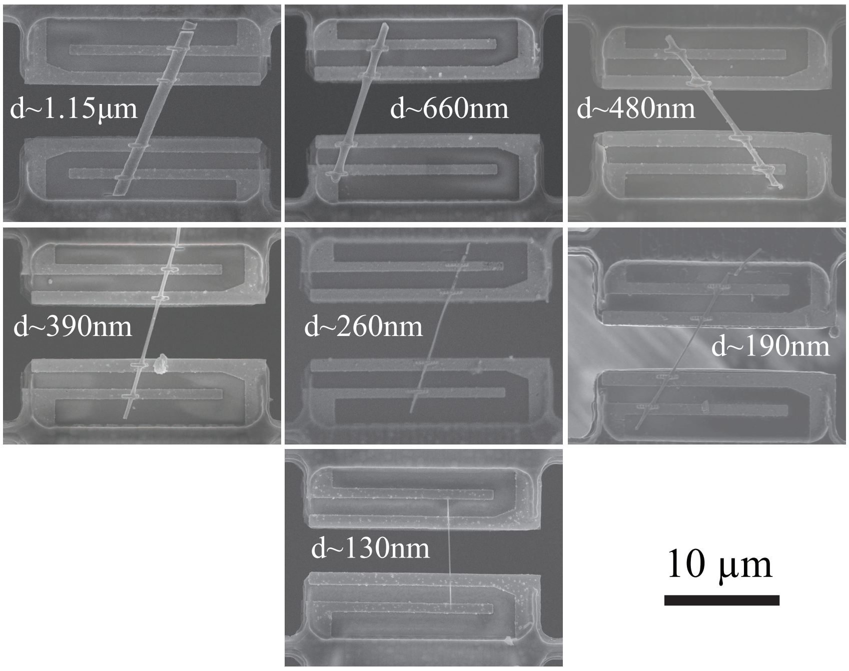

Figure S 3: SEM images of SnSe nanowires aligned on specialized micro devices for diameters between 1.15 μm to 130 nm

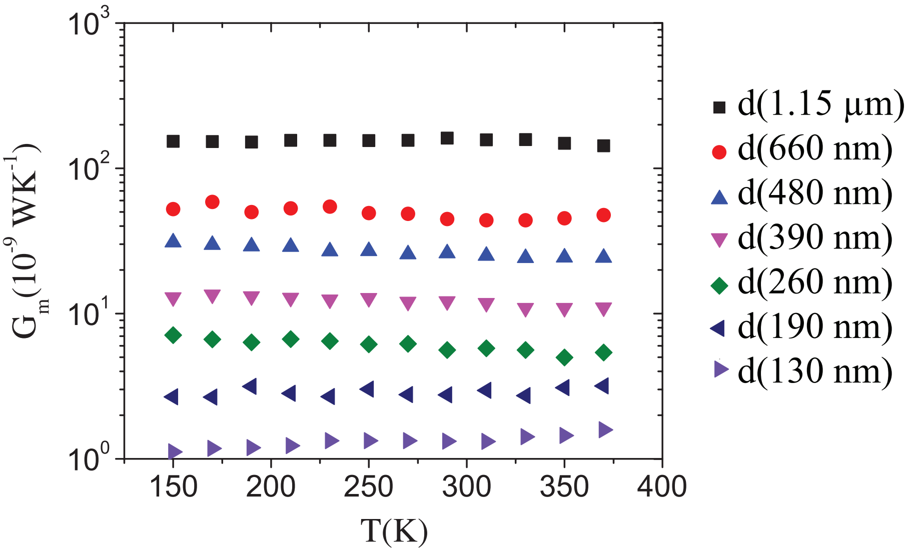

Figure S 4: Measured total thermal conductance (Gm) versus temperature (T) for seven nano- and micro-wires

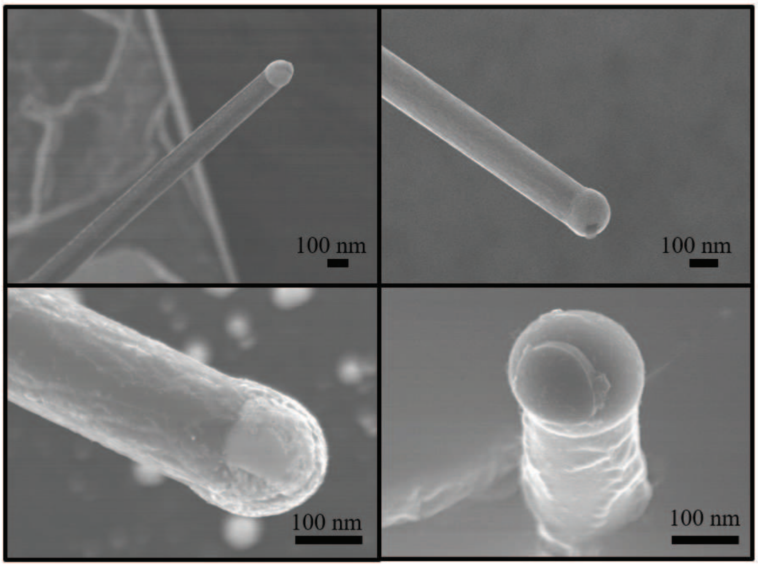

Figure S 5: SEM images of the SnSe nanowires on the substrate. The cross section suggests rounded shape

Why Bulk calculations?

Using the Vienna ab-initio simulation package (VASP), projected density of states calculation was performed to demonstrate that surface electron states in a 100 nm SnSe sample contribute less than $\sim 1\%$ of the total states. A (1 1 1) slab was analyzed in the orthorhombic structure of the SnSe crystal, holding a total of 18 layers and 5nm thick slab. As in the main work, a many-body GW approximation was implemented with the

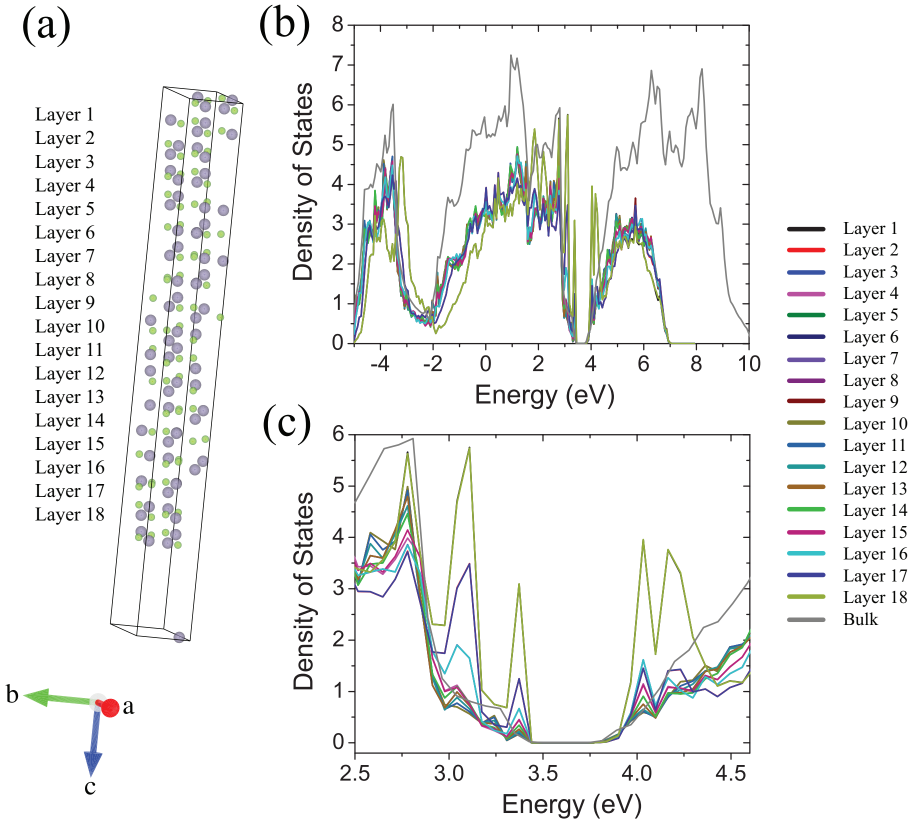

Figure S 6: (a) Supercell Crystal structure of the low temperature Pnma phase SnSe with 18 layers. Gray balls are Sn atoms and green are Se atoms. (b) Calculated Density of States per layer as a function of the chemical potential for modeled nanowire with 5nm in diameter (c) is the same as (b) with different scales

projector augmented wave (PAW) scheme, and the generalized gradient approximation of Perdew, Burke and Ernzerhof (GGA-PBE) for the electronic correlation functional. A gamma centered grid with $k-$point meshes of $3 \times 9 \times 1$ and a cutoff energy of 450 eV was chosen for the self-consistent calculation. The k-point mesh was increased to $5 \times 15 \times 1$ for the non-self-consistent calculation holding the same cut off energy.

The contribution of states per layer is presented in the projected density of states calculation (Fig S6). Results clearly demonstrate the little share that surface states present in the inner 4th to 14th layers that already resemble the bulk density of states. The synthesized nanowires have diameters larger than 100 nm, these results justify the use of 3D periodic Density of States configuration to calculate the electron energies of the measured SnSe nanowires

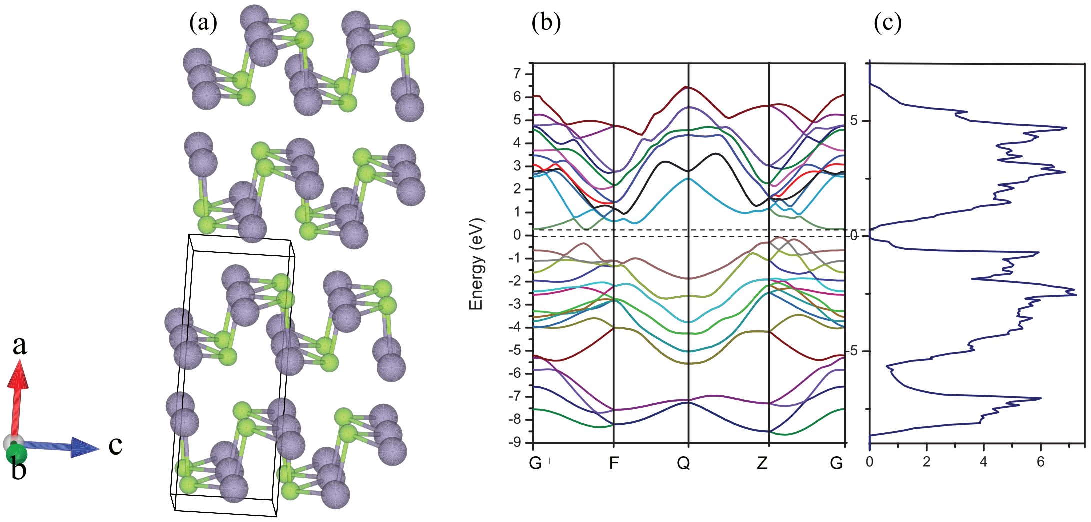

Figure S 7: (a) Crystal structure of the Pnma phase of SnSe. The primitive cell is shown as a black line. Gray larger balls represent Sn atoms and green smaller balls are Se atoms. (b) The calculated band structure and (c) the total density of states, using to calculate electrical thermal conductivity (κ_e)

Modified Debye-Callaway model

We fitted $\kappa_{ph}(T)$ using a modified Debye-Callaway model [1], with contributions due to impurities, point defects, umklapp and surface boundary phonon scattering events. Normal scattering contribution goes linear with frequency and does not contribute significantly to the total thermal conductivity. In general, $\kappa_{ph}(T)$ can be expressed as:

$$\kappa_{ph}(T) = \frac{k_B}{2\pi^2 v_s}\left(\frac{k_B T}{\hbar}\right)^3 \int_0^{T_D/T} \frac{x^4 e^4}{\tau_{ph}^{-1}\left(e^x - 1\right)^2} dx \quad (1)$$

with:

$$\tau_{ph}^{-1} = \tau_I^{-1} + \tau_D^{-1} + \tau_U^{-1} + \tau_B^{-1} = A\alpha^4 T^4 x^4 + RTx + B\alpha^2 T^3 x^2 e^{-\frac{T_D}{T}} + \frac{v_s}{L} \quad (2)$$

Where $x = \hbar\omega/(k_B T)$ is the reduced phonon frequency, $\hbar$ is the Planck constant, k_B is the Boltzmann constant, v_s = 1746 m/s is the speed of sound in SnSe, T_D = 49K is the Debye temperature for SnSe and T is the absolute temperature. The chosen values for v_s and T_D for the [111] growth direction of our samples correspond to the average of the three crystal orientations taken from Zhao et. al. calculations [2]. For the [111] direction the average weights the sound velocities along each crystal axis equal ($\frac{1}{3}$ each). The fitting parameters are A for impurities, R dislocations, B for umklapp process and L is the mean free path due to surface boundary scattering. Here we assign L to the measured diameter of the nanowire. In order to stress the contribution of the phonon surface scattering to the reduction of the thermal conductivity in SnSe nanowires, we

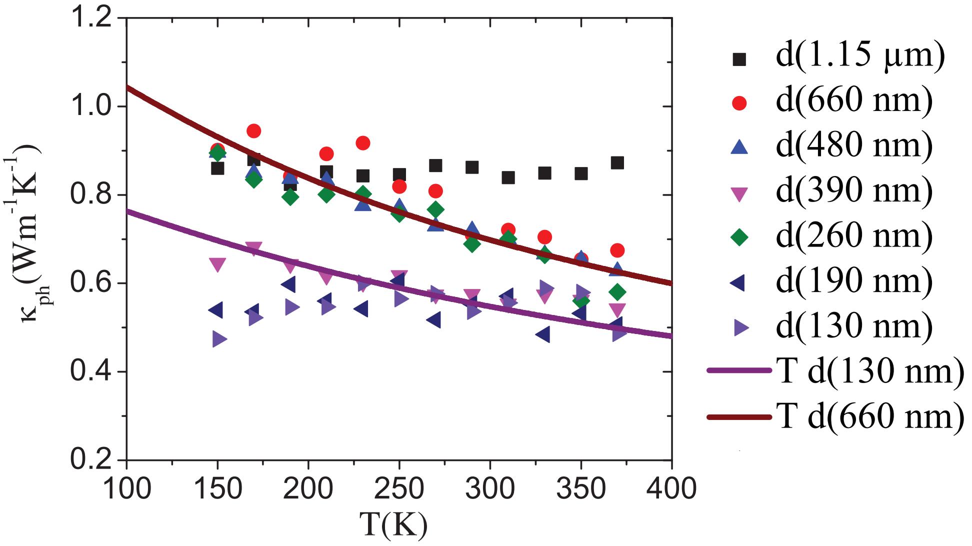

Figure S 8: The measured lattice thermal conductivities (κ_{ph}) for each nanowire. The calculated κ_{ph} for two diameters (L_1 and L_2) are shown with continuous lines

maintained constant $A = 1 \times 10^{-42}\ s^3$, $R = 7.5 \times 10^8\ s^{-1}K^{-1}$, $B = 5 \times 10^{-18}\ sK^{-1}$ in all cases and calculated the thermal dependence of κ_{ph} for two diameters: $L_1 = 130$nm and $L_2 = 660$nm. Figure S8, shows an overall match between the fitting from the modified Debye-Callaway model and the experimental data for larger and smaller diameters giving some role to the phonons surface scattering to the thermal conductivity within the range of diameters used in this study, measuring 0.55 $\mathrm{Wm^{-1}K^{-1}}$ at $T = 300$K in the $d = 130$nm nanowire. Multilayered materials with layer thickness $\sim$ 30nm reported lower thermal conductivities [3] that anticipates a further reduction of κ in nanowires with lower diameters.

References

1. Callaway, J.,. Phys. Rev. 1959 113(4),1046.

2. Zhao, L.D.,. Nature 2014, 508(7496), 373-377.

3. Serrano-Sánchez, F.,. Appl. Phys. Lett. 2015, 106(8), 083902
